# A reconciliation of genome-scale metabolic network model of Zymomonas mobilis ZM4

**DOI:** 10.1038/s41598-020-64721-x

**Published:** 2020-05-08

**Authors:** Hoda Nouri, Hamideh Fouladiha, Hamid Moghimi, Sayed-Amir Marashi

**Affiliations:** 1 Department of Microbial Biotechnology, School of Biology, College of Science, University of Tehran, Tehran, Iran; 2 Department of Biotechnology, College of Science, University of Tehran, Tehran, Iran

**Keywords:** Metabolic engineering, Computer modelling, Metabolic engineering

## Abstract

*Zymomonas mobilis* ZM4 has recently been used for a variety of biotechnological purposes. To rationally enhance its metabolic performance, a reliable genome-scale metabolic network model (GEM) of this organism is required. To this end, we reconstructed a genome-scale metabolic model (*i*HN446) for *Z. mobilis*, which involves 446 genes, 859 reactions, and 894 metabolites. We started by first reconciling the existing GEMs previously constructed for *Z. mobilis* to obtain a draft network. Next, recent gene annotations, up-to-date literature, physiological data and biochemical databases were used to upgrade the network. Afterward, the draft network went through a curative and iterative process of gap-filling by computational tools and manual refinement. The final model was evaluated using experimental data and literature information. We next applied this model as a platform for analyzing the links between transcriptome-flux and transcriptome-metabolome. We found that experimental observations were in agreement with the predicted results from our final GEM. Taken together, this comprehensive model (*i*HN446) can be utilized for studying metabolism in *Z. mobilis* and finding rational targets for metabolic engineering applications.

## Introduction

Bioethanol production has received increasing attention as a sustainable replacement for fossil fuels. *Zymomonas mobilis*, as an ethanologenic Gram-negative bacteria, possesses desirable characteristics because of its unique usage of the Entner-Doudoroff (ED) pathway. Compared to other ethanologenic microorganisms, *Z. mobilis* shows a higher glucose uptake rate and also higher yields of ethanol production. These characteristics are considered as the prerequisites for an industrially effective ethanol production process^1,2^.

In addition to ethanol, *Z. mobilis’* metabolism consists of endogenous metabolic pathways that produce other industrially notable products including levan, sorbitol, glycerol, gluconic, succinic, lactic and acetic acids^3^. Levan is an important biopolymer has versatile applications in pharmaceutical, food and cosmetic industries^3^. Levan is a homo-exopolysaccharide which is composed of d-fructose monomers linked by β (2 → 6) bonds. Levansucrase (EC 2.4.1.10) is involved in the polymerization of fructose units, using sucrose as substrate^4^. All these characteristics suggest that *Z. mobilis* has significant potential for the production of an extended range of valuable biochemical compounds for industrial biotechnology applications^3,4^

Recently, manipulation and improvement of *Z. mobilis* have been reported via systems biology and computational modeling, evolutionary engineering, genome editing, omics technology (genomics, transcriptomics, proteomics, metabolomics) and synthetic biology^3–5^. In addition, diverse metabolic engineering approaches have been used to redesign the new metabolic pathways in *Z. mobilis* such as deletion of competing pathways, insertion of new genes to increase substrate ranges, improve tolerance to extreme process conditions, ethanol toxicity and lignocellulosic hydrolysate inhibitors^4,6^.

In 2005, the primary genome sequence of *Z. mobilis* ZM4 was published and its annotation upgraded in 2009^7,8^. Recently, complete chromosome along with four native plasmids were sequenced in ZM4. These plasmids were in the size range of 32 to 39 kb and containing about 150 predicted ORFs^9^. In recent decades, *in silico* genome-scale metabolic models (GEMs) have introduced novel avenues for systems-level redirection of metabolic fluxes, and consequently, rational strain improvement^10,11^. *Z. mobilis* is generally considered as a suitable platform for genetic engineering because of its GRAS (Generally recognized as safe), genome size, and efficient fermentation performance during a low biomass production rate^2^. Thus, purposeful metabolic engineering modifications can potentially be done in this organism.

In the present study, our goal is to develop an updated GEM for *Z. mobilis* ZM4. Several studies have previously reported core metabolic model^12^ or GEMs^13,14^ for *Z. mobilis* ZM4. Furthermore, a GEM for a related strain, *Z. mobilis* ZM1 was reported^15^. However, due to the existing limitations and inconsistencies of those models, we decided to reconstruct an updated, reconciled and comprehensive *Z. mobilis* ZM4 GEM. None of the previously-published *Z. mobilis* GEMs were made in formats that are ready for simulation testing. We tried to check and find the required reactions to make a working model because none of these GEMs were able to produce biomass. The process of biomass correction, which was done for both GEMs (ZmoMBEL60 and *i*ZM363), was in fact non-negligible. The previous GEMs use different metabolite names and different metabolic subnetworks. In addition, in some cases, multiple names of metabolites and reaction abbreviations were included. Both of the previously-published GEMs were constructed in the same year. Interestingly, their metabolic coverage, biochemical evidence and GPRs are not consistent and differ in some cases. The core metabolic network was improved mainly based on literature evidence, but new enzyme-related together with new reactions and GPRs were not necessarily included.

**Figure 1:**
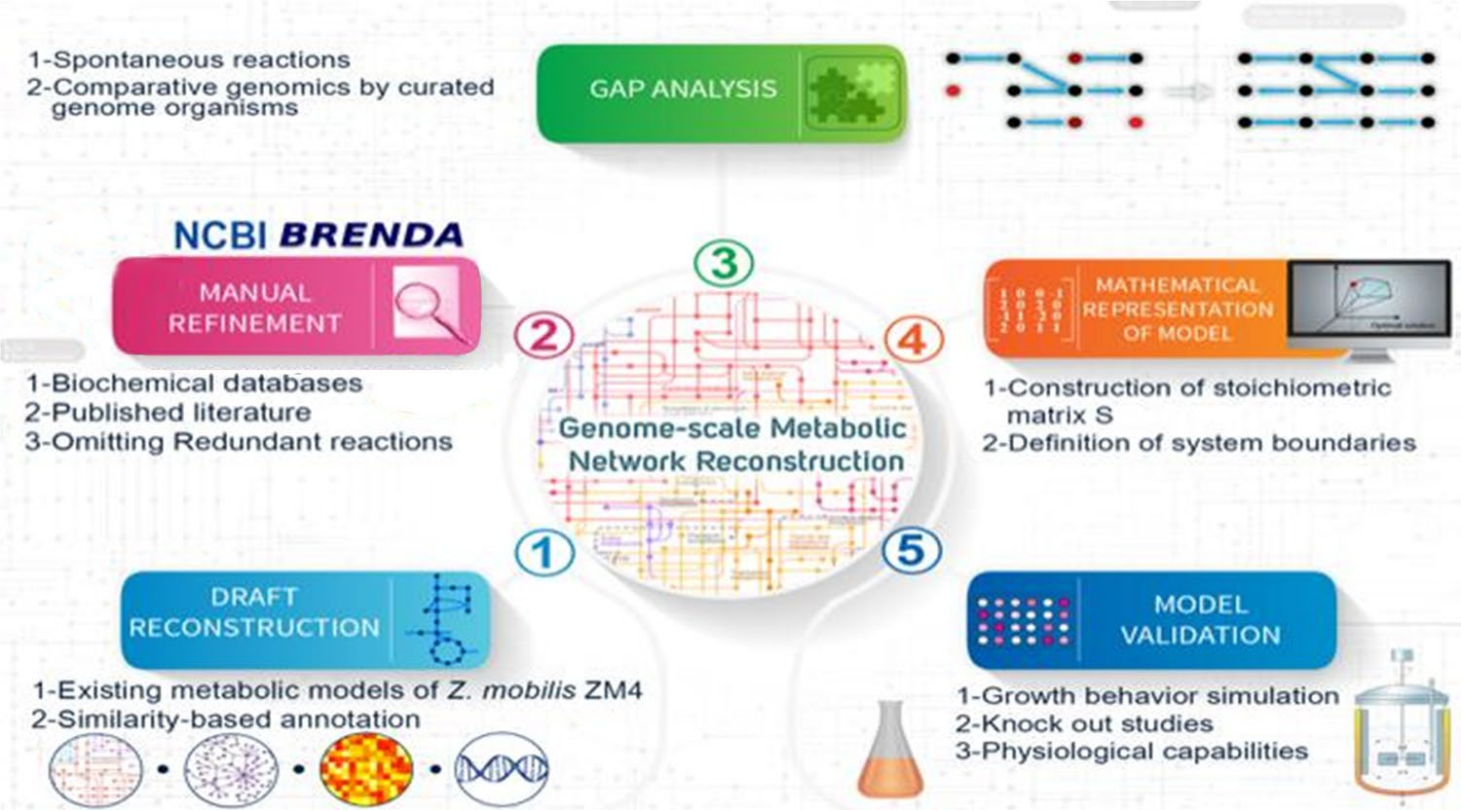
Schematic representation of the reconciliation workflow which was used for the reconstruction of *i*HN446, the updated genome-scale metabolic model of *Z. mobilis* ZM4.

Consequently, as more data about physiology, biochemistry and genetics was available for *Z. mobilis* ZM4, it is necessary to update the GEM of this organism by retrieving the relevant data from literature and publicly available databases. We did this update with additional manual curation (Fig. 1).

## Materials and Methods

### Metabolic network draft reconstruction

The three previous *Z. mobilis* GEMs and one *Z. mobilis* core metabolic network were compiled for the reconstruction of our initial draft model^12–14^. In the first step, the list of all metabolic network components (including genes, proteins, reactions and metabolites) involved in the previous *Z. mobilis* models were collected. As a key bottleneck in metabolic models reconstruction, incompatibilities in the content of metabolic models such as multiple metabolites names, reaction abbreviations and stoichiometric errors were fixed. The redundancies were then verified and components with multiple/inconsistent IDs were detected and removed. Consequently, in our model, a consistent and unique ID (compatible with the KEGG database) was assigned to each protein, reaction and metabolite. obtained and manually checked for association with enzyme and/or reactions in KEGG.

In the next step, for each enzyme, all associated genes as well as all possible reactions were determined using KEGG^16^ and BRENDA^17^ databases. Furthermore, the list of all genes present in the *Z. mobilis* ZM4 genome was obtained and manually checked for association with enzyme and/or reactions in KEGG. Next, new genes that were not present in previous GEMs were identified and added to the model in order to improve gene-protein-reaction association correctness. In some cases, for confirming the functions of genes, sequence similarity with *Escherichia coli* was used. Non-gene-associated reactions, including spontaneous reactions and literature-based metabolic activities were also manually curated and added to the model. All instances of mass imbalance and stoichiometric errors were then found and fixed. The reversibility types of reactions were assumed to be the same as those of the template models. In cases of discrepancies among the templates, the reversibility types of reactions were determined based on BioCyc^18^ and KEGG. Exchange reactions were retrieved from the relevant literature and TransportDB^19^.

### Gap detection

No-production and/or no-consumption metabolites often represent knowledge gaps in metabolic networks^20^. We identified such metabolites in the draft model. Then, in an iterative procedure, we tried to find a minimal set of biologically relevant reactions that were able to fix the gaps using fastGapFill^21^. Additionally, GapFind^22^ algorithm was used to find further dead-end metabolites and blocked reactions. Some reactions were proposed by these reaction-addition algorithms. We tried to identify those that are biologically reasonable by using genomic and biological evidences to avoid overfitting model. We also evaluated the accuracy of identifying filled gaps manually when possible, by finding missing reactions when compared to the GEMs in BiGG^23^ database. In some cases, direction of reactions in model was changed based on biological knowledge. Some spontaneous reactions that were previously confirmed experimentally in *E. coli* were also added to our network. Finally, as there are several orphan reactions in each GEM, we analyzed blocked reactions of the *E. coli* model^24^. So, more biological discoveries should be done to fill these gaps.

### Constraint-based modeling: Flux Balance Analysis

In a metabolic network model, there are prevalent interdependencies among reaction fluxes due to stoichiometry, reversibility and capacity constraints of metabolism. Flux balance analysis (FBA) is used for optimizing a pre-defined objective function in the specified metabolic constraints. Fundamentals of FBA have been explained previously^10^. Unless stated otherwise, in the present work, maximization of biomass production rate is considered as the objective function. All analyses were performed using COBRA Toolbox v2.0^25^. GLPK was applied as the linear programming solver to perform FBA.

For all *in silico* simulations based on carbon source usage, the lower bound of the carbon source uptake rate was considered as −10 mmol gDCW^−1^ h^−1^. Unless stated otherwise, for all reactions, the upper bound was assumed to be +1000 mmol gDCW^-1^. The lower bounds of the irreversible and reversible exchange reactions were set 0 and −1000 mmol gDCW^−1^ h^−1^, respectively.

For simulation of the aerobic and anaerobic conditions, −1000 and 0 mmol gDCW^−1^ h^−1^ was considered for oxygen exchange reaction, respectively. ATP hydrolysis and synthesis flux values were set to −10 and 50 mmol gDCW^−1^ h^−1^ respectively, based on Motamedian *et al*. results^15^. The lower bound of amino acid exchange reactions was set to 0.1 mmol gDCW^−1^ h^−1^ when the medium was consisting of yeast extract. Non-growth-associated (NGAM) and growth-associated maintenance (GAM) were same as Widiastuti *et al*. results^14^.

### Model validation

We validated our model through four major approaches. Batch fermentation of *Z. mobilis* ZM4 grown on different culture media in aerobic and anaerobic conditions was done to examine whether our model correctly predicts the growth rate patterns. Moreover, a comprehensive literature search was performed to find studies of genetic engineering and gene knockout on *Z. mobilis*. Furthermore, gene essentiality analysis was done to check the accuracy of the model prediction. In order to further evaluate the accuracy of our model, the profile of differentially expressed genes in aerobic vs. anaerobic conditions was investigated. Then, the transcriptionally-changed enzymes were used to qualitatively validate the oxygen-related changes in the predicted fluxes^26^. Additionally, Biolog GN2 MicroPlate™ was used in order to profile growth phenotypes of *Z. mobilis* ZM4 on diverse carbon sources. According to the standard protocol, a colony of the bacterium was inoculated on 5% Sheep Blood that is supplemented to a Biolog Universal Growth medium for an overnight at 37 °C. The bacteria were then removed using a sterile swab and suspended in GN/GP Inoculating Fluid to the definite turbidity. Then, 150 µl aliquot of cell suspension was inoculated into each of the 96 carbon source-containing wells. In positive cases, i.e., where *Z. mobilis* can grow, by making a redox potential, a purple color will appear because of reduced tetrazolium dyes. Additionally, a previously reported list of biochemical results was used to validate the predictions^27^.

### Fermentation experiments with *Z. mobilis*

*Z. mobilis* ZM4 ATCC 31821 was cultured in RM medium (Glucose 20 g/L, KH_2_PO_4_ 2.0 g/L, Yeast Extract 10 g/L) at 30 °C^26^. For ethanol production, fermentation was carried out in either of two different media, namely, standard RM and modified RM (Glucose 20 g/L, Yeast Extract 10 g/L, KH_2_PO_4_ 2 g/L, MgSO_4_·7H_2_O 1 g/L, (NH_4_)_2_SO_4_ 1 g/L)^14^. For levan production, the culture medium (SRM) contained sucrose 100 g/L, Yeast Extract 10 g/L, and KH_2_PO_4_ 2.0 g/L. Cells were grown for preculture preparation in 50 mL medium in a 250 mL Erlenmeyer flask. Shaking and incubation were done at 150 rpm and at 30 °C, respectively. Afterward, anaerobic condition for ethanol production was achieved by culturing in sealed bottles filled with 50 ml of culture medium. For aerobic ethanol or levan production, 20 mL medium was used in a 100 mL Erlenmeyer flask. Samples were withdrawn throughout fermentation at different intervals for further analysis. All experiments were repeated at least three times with three replicates under each of the conditions.

### Analytical methods

During fermentation, growth was checked spectrophotometrically by measuring optical density at a wavelength of 600 nm (Rayleigh UV-1601). Glucose content of each sample was quantified by using an enzymatic kit that is based on glucose oxidase activity^28^. Gas chromatography was applied to determine the ethanol content of each distilled medium. Levan extraction was performed by absolute ethanol and dialysis (MWCO 14000 Da). Quantitative analysis of produced levan was done by phenol-sulfuric acid method and estimated as fructose units^29^.

## Results and Discussion

### Genome-scale metabolic network of *Z. mobilis* ZM4

Figure 1 illustrates a summary of our GEM reconstruction procedure. Briefly, the three previously-published *Z. mobilis* ZM4 GEMs were unified and reconciled. Furthermore, in order to construct our comprehensive GEM, several new genes, enzymes, reactions and metabolites were added to the previous models based on database searches and literature evidence.

Different data sources were used for model compilation, including (i) BiGG, BioCyc, KEGG, BRENDA, and TransportDB databases, (ii) genomic sequence of *Z. mobilis* ZM4 for analyzing sequence similarity by using BLAST and NCBI, (iii) previously-published *Z. mobilis* GEMs for gathering all reactions and genes, and other literature-based evidence that were reported earlier. Two models of *Z. mobilis* were genome-scale, while the other one is limited to central metabolism, in which the focus was mostly on biochemical and enzymatic evidence. Consequently, we gathered all these pieces of information for gaining a comprehensive draft model from previous researches. Furthermore, in some cases that dead-end metabolites or blocked reactions were found, *E. coli*^24^ and *B. subtilis*^30^ GEMs were used, as high quality bacterial templates, to ensure the presence of spontaneous reactions. Then we checked the presence of these reactions in *Z. mobilis*, as well.

Since the two GEMs had different identifiers for metabolites, we searched for equivalent reactions and removed redundancies. Furthermore, whenever necessary, we fixed the discrepancies in stoichiometries, reversibility type of reactions and gene-protein-reaction associations. We then analyzed the orphan (*i.e*., non-gene-associated) reactions to check their reliability, followed by reaction gap filling. Altogether, 188 reactions were added from the KEGG database to refine our model. In this procedure, a considerable number of gaps were filled, and several blocked reactions were unblocked.

A recent report has presented comprehensive data on the sequences and annotations of plasmid-related genes in *Z. mobilis* ZM4 (including pZM32, pZM33, pZM36 and pZM39)^9^. However most of these genes have non-metabolic functions. Their function was characterized in the field of phage structure proteins, toxin-antitoxin proteins, membrane-associated transporters and transcriptional regulators, as well as several hypothetical proteins. It should be noted that certain plasmid-related genes with metabolic functions had the same enzymatic activity as those genes which were present in our GEM. Finally, we updated our GEM and added some reactions based on these plasmid sequence revisions. The final GEM, which will be referred to as *i*HN446, includes 446 genes, 859 reactions, and 894 metabolites. The *i*HN446 model, in XLS format, which includes the complete list of reactions and metabolites, can be found in File S1. We also provide the model in SBML format (File S2).

**Figure 2:**
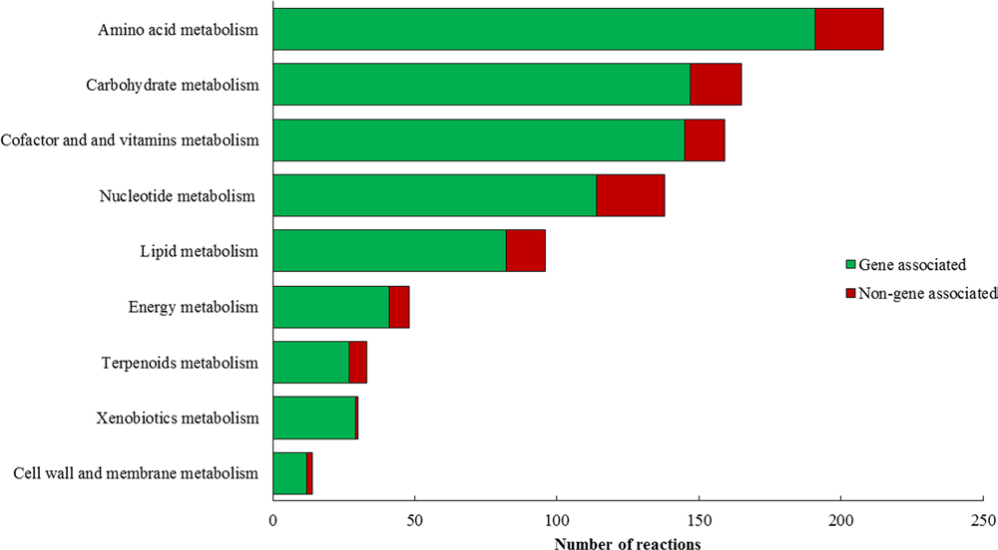
The portion of gene associated reactions in each of nine functional categories. Non-gene-associated reactions in each subsystem are shown as the black part of each bar.

Figure 2 summarizes an overview of the number of non-gene associated and gene-associated reactions in each of the pathways in *i*HN446. For genetic engineering purposes, the gene-to-protein associations are of central importance. Our results suggest that the reactions included in *i*HN446 are reasonably reliable, as most of the reactions in the model are associated with their corresponding metabolic genes (Fig. 2). One can obviously observe that amino acid metabolism had the most reactions in the metabolic network. Also, previous flux coupling results had confirmed the important role of this pathway in ethanol production and the growth of organism^14^.

We then compared *i*HN446 with all previously-published *Z. mobilis* metabolic network models (Table 1). In the chronological order, the two *Z. mobilis* ZM4 GEMs were reconstructed by Widiastuti *et al*. and Lee *et al.*^13,14^, respectively. Then, a core metabolic network model of *Z. mobilis* was released by Pentjuss and coworkers^12^. Finally, Motamedian *et al*. published another GEM for a related strain, namely *Z. mobilis* ZM1^15^.

**Table 1:** The characteristics of different genome-scale metabolic models of *Z. mobilis*.

	*i*HN446 (this study)	Core model^12^	ZmoMBEL601^13^	*i*ZM363^14^	*i*EM439^15^
No. of gene-associated reactions	689	43	493	414	585
No. of non-gene-associated reactions	170	12	70	190	107
No. of included genes	446	41	348	363	439
Gene coverage in the model *	22.3%	2%	17.4%	18.2%	22.7%
No. of metabolic reactions	859	55	563	604	692
No. of transport reactions	73	21	37	143	71
No. of cytoplasmic metabolites	821	62	578	605	649
No. of extracellular metabolites	73	24	31	99	37

*Percentage of total gene coverage based on overall genes in *Z. mobilis* strains (*i*HN446, Core model, ZmoMBEL601 and *i*ZM363 consist of 1998 genes. *i*EM439 consists of 1929 genes)

Total gene coverage of model is 22.3% in *i*HN446, while ZmoMBEL601 and *i*ZM363 models each have 17.4% and 18.2% coverage, respectively. Since *Z. mobilis* ZM1 genome includes fewer genes in comparison to *Z. mobilis* ZM4 (i.e., 1929 vs.1998), the gene coverage of *i*EM439 should not be compared with the gene coverage of other GEMs.

In *i*HN446, the gene-associated reactions account for 80% of all non-transport reactions. In comparison, ZmoMBEL601, *i*ZM363 and *i*EM439, 87%, 68% and 84% of the non-exchange reactions are gene-associated, respectively.

**Figure 3:**
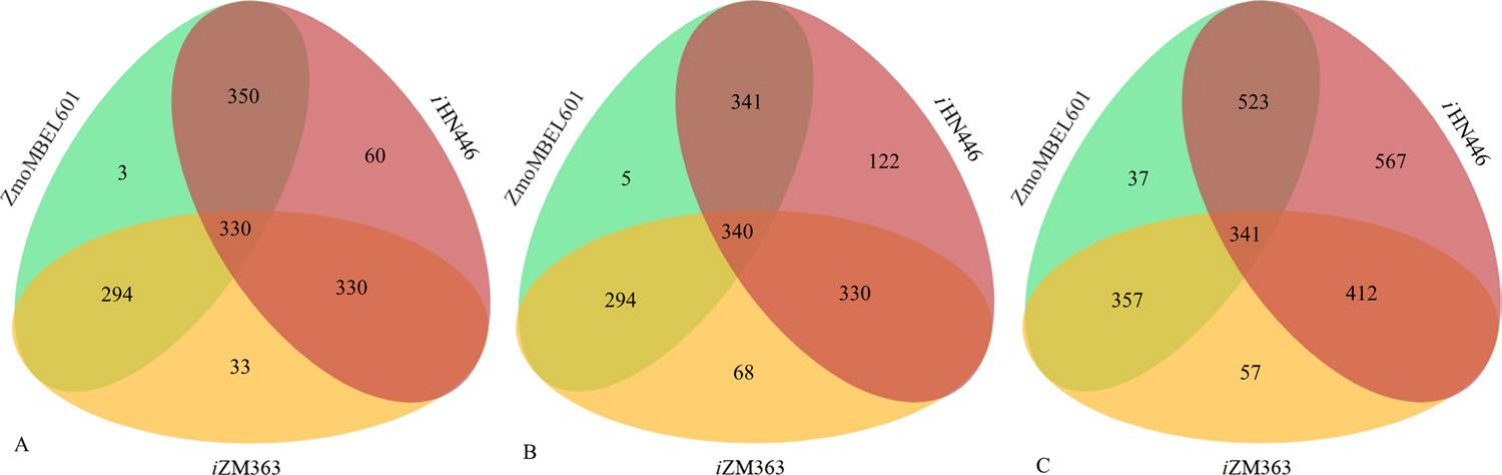
Comparison of genes (**A**), enzymes (**B**) and reactions (**C**) shared by *i*HN446, ZmoMBEL60 and *i*ZM363.

As expected, our reconciled model includes a considerable number of unique metabolites, genes and reactions (and more specifically, gene-associated reactions). The number of unique (and also shared) genes, enzymes and reactions are depicted in Fig. 3 and Table S3. In comparison to previous GEMs, our new model has 567 new reactions, 122 new enzymes and 60 new genes. These data show that the upgraded model has significant improvements in metabolic coverage. Among these 567 updates, reactions of energy, lipid, cofactor and vitamin metabolism, together with the metabolism of secondary metabolites are corrected. Comparison of previous models revealed that there are 357 reactions common between the two models, while 57 and 37 reactions were unique to *i*ZM363 and ZmoMBEL60, respectively. In addition, *i*ZM363 has 63 more unique enzymes and 30 more unique genes in comparison to ZmoMBEL60.

Although a great ratio of *i*HN446 is shared by the previous models, we excluded genes, enzymes or reactions from our model for which we did not find adequate evidence confirming their existence. Additionally, in certain cases, changes in the biochemical data or genomic annotations might have resulted in the inconsistencies (Table S4).

Taken together, we propose that our model is upgraded in various parts in comparison to previous models of *Z. mobilis* ZM4:

I. Conversion of each GEM to a machine-readable format was required. In the first step, after we converted these GEMs to SBML format, COBRA toolbox was used for analysis, but none of these GEMs were able to synthesize biomass. This makes simulation analysis difficult because the conversion of a GEM to a mathematical format is required for biological studies. Note that addition of required reactions may inevitably result in a slightly different model in comparison to the original version of the models.
II. In the present model, we assigned KEGG metabolite and reaction identifiers to most of the metabolites and reactions in the model, because different or multiple metabolite names were included in the each of the previous GEMs.
III. The charged forms of metabolites are not presented in the previous GEMs. Also, both GEMs ignored mass and electrical charge balance.
IV. Although in the same year both of the previous GEMs of *Z. mobilis* ZM4 were constructed, they showed different gene-protein-reaction coverage. In addition, in some cases, metabolic and biochemical data were considered differently.
V. In ZmoMBEL60 model, although yeast extract is an ingredient of the culture medium, neither the amino acids nor the trace elements were included in the model (based on the present exchange reactions). Consequently, simulation results are not performed in the same condition as experimental ones.
VI. When we tried to build a biomass-producing model, ZmoMBEL60 was dependent on NMN entrance to the cell for biomass production in simulation tests. Besides, some exchange reactions for some metabolites like, NAD^+^, AMP and NMN are considered to be present in the model. Such a dependency to the uptake of NMN (and other metabolites) does not occur in reality.
VII. In *i*ZM363 model, phospholipid and cardiolipin were not able to be produced, and consequently, no biomass production was obtained. Therefore, we decided to allow these two biomass constituents to enter the network by adding their exchange reactions and checking the biomass production. In the culture condition proposed in Widiastuti *et al*. article, biomass production was simulated. However, neither in the original condition nor in this new biomass formulation, a non-zero growth rate was seen. We tried in an iterative process to find essential metabolite(s) for enabling biomass production. In this case we found that isoleucine, glycine and S-adenosyl-l-methionine supplementation is vital for biomass production.
VIII. In the core metabolic network, Pentjuss *et al*. included a number of biochemical reactions with experimental evidence, mainly on NAD(P)H balance, which is necessary for stoichiometric balance. Also, a number of important changes were made in respiratory chain and catabolic genes in the central carbon metabolism, which were not incorporated in previous reconstructions. Although this model was improved mainly based on literature evidence, new enzyme-related changes, together with new reactions and GPRs were not necessarily included. Basically, this model cannot be considered as a GEM with comprehensive list of genes, reactions and metabolites. The improvements suggested by Pentjuss *et al*. are included in our GEM.

### Model validation

#### Carbon-Nitrogen-Sulfur utilization phenotypes

In case of carbon sources, we used both qualitative and quantitative studies to determine the metabolic capabilities of *Z. mobilis*. The Biolog analysis was used to qualitatively study the utilization of several different carbon sources. Additionally, consumption of glucose, fructose and sucrose by *Z. mobilis* are further analyzed quantitatively. In each case, growth rate, together with ethanol and/or levan production were measured in aerobic and anaerobic conditions.

A high-throughput 96-well Biolog phenotypic test was performed to profile carbon usage of *Z. mobilis* ZM4. Among the carbon sources available on the Biolog MicroPlate™, we observed carbon source utilization only in two cases, namely fructose and glucose. Although sucrose utilization has been reported in *Z. mobilis*, our Biolog phenotypic result was negative, which is consistent with the previously reported *Z. mobilis* growth results^27^. Since *Z. mobilis* is known to be able to utilize sucrose for growth and levan production^31^, we carefully studied sucrose-dependent growth. We used broth culture and plates supplied by sucrose as carbon source. In both of the solid and liquid cultures, the growth of *Z. mobilis* was observed.

**Figure 4:**
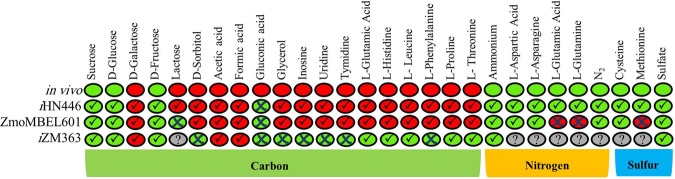
Results of *in silico* simulations and experimental experiments based on growth on different carbon, nitrogen and sulfur sources (Growth/True (Green☑), Growth/False (Green☒), No-Growth/True (Red☑), No-Growth/False (Red☒), Non-tested (?).

In order to computationally simulate the carbon source utilization capabilities and comparing the results obtained with previous *Z. mobilis* GEMs, we used FBA. As it is presented in Fig. 4, the exact accordance between modeling and experimental data were seen in all three models for the three carbon sources as mentioned above. In ZmoMBEL60 model, no transport reaction was included in the model for other carbon sources. Consequently, we added transport reactions in these cases. In ZmoMBEL60 model, lactose and gluconic acid were incorrectly predicted to be utilized by *Z. mobilis*. In *i*ZM363 model, sorbitol, gluconic acid, glycerol, inosine, uridine, thymidine and phenylalanine were incorrectly predicted to be consumed by *Z. mobilis*. Also, *i*HN446 mistakenly predicts the gluconic acid utilization as the carbon source. Therefore, a comparison of modeling results with experimental data confirms the precision of our model.

In case of nitrogen and sulfur sources, we used the previously-published data by Bochner *et al*. for model validation^27^. Simulations suggest that *i*HN446 is able to successfully predict l-asparagine, l-aspartic acid, l-glutamine and ammonia consumption as the nitrogen source. ZmoMBEL60 model was able to predict the consumption of ammonia as the nitrogen source. Although, since yeast extract (including amino acids) is not included in the *in silico* growth medium, simulation on different amino acids as nitrogen source was not possible. In this case, we included transport reactions of nitrogen sources to model. Simulation proposed that ZmoMBEL60 is able to predict l-asparagine and l-aspartic acid, correctly. In contrast, l-glutamine was mistakenly consumed by *Z. mobilis*. In addition, in *i*ZM363 model, isoleucine and glycine should be added to the growth medium to enable biomass synthesis. Therefore, these amino acids are essential and are consumed, and hence, they can provide the required nitrogen for *Z. mobilis*. Consequently, it is not possible to simulate the necessity of any nitrogen sources.

Sulfate, l-cysteine and l-methionine utilization were correctly predicted to be used by *i*HN446 as the sulfur source. ZmoMBEL60 model, in contrast, could only predict the sulfate consumption. In *i*ZM363 model, S-adenosyl-l-methionine supplementation was necessary for growth, and therefore, one could not simulate the usage of sulfur sources. Taken together, simulation results of the *i*HN446 model showed an improved consistency with the experimental results, compared to the two previously-published GEMs (Fig. 4).

### Batch fermentation characteristics

The growth patterns of *Z. mobilis* in the two different growth media (see Materials and Methods) were determined experimentally. Then, these data were compared with *in silico* simulation results obtained by FBA using the biomass production rate as the objective function. In RM and modified RM media, we found the model predictions to be in agreement with the experimental data in all conditions. By performing FBA in the conditions of culture media, the growth rate was determined as 0.136 h^−1^ and 0.5 h^−1^, respectively, which is comparable to the experimentally measured growth rate (0.154 ± 0.03 in RM and 0.39 ± 0.04 in modified RM). The ethanol content was 6.28 ± 0.8 g/L and 7.38 ± 0.51 in RM medium in aerobic and anaerobic conditions, respectively. Additionally, in the modified RM medium, ethanol quantities were 7.23 ± 0.36 g/L in aerobic and 8.6 ± 0.48 g/L in anaerobic fermentation. Furthermore, we ran FVA and analyzed the maximum and minimum flux rates of reactions in which ammonium is involved. As expected, in modified RM with ammonium supplementation, these reaction fluxes changed to zero or decreased significantly. It should be noticed that there are 20 reactions in the *i*HN446 model that produce or consume ammonium. So, our model could reveal the effect of ammonium addition. Based on FVA results under two different culture media, evaluation of fluxes of amino acid production metabolism revealed that in modified RM amino acid metabolism is considerably less active. These observations confirmed that the addition of ammonium to medium reduces the dependence of *Z. mobilis* to amino acids because ammonium could go through different pathways. In conclusion, the addition of ammonium and flux changes agrees with the reported experimental results^32^.

In the simulation with sucrose as the carbon source, the predicted results confirmed our experimental outcomes. The calculated *in silico* and experimental growth rate were 0.136 h^−1^ and 0.168 ± 0.03 h^−1^, respectively. Measurement of levan production showed 0.6 ± 0.07 g/L of product formation under the experimental condition. Furthermore, we used FVA to assess the range of levansucrase flux. FVA results confirmed that this pathway was functional, as the maximum and the minimum fluxes of the reaction were 5 mmol gDW^−1^.h^−1^. Moreover, pathways of sucrose and fructose metabolisms were active when sucrose was added as a carbon source to the medium. Minimum and maximum flux of d-sorbitol dehydrogenase were −15.25 and 20 mmol gDW^−1^.h^−1^, respectively as fructose is needed for sorbitol production. Also, glucose-fructose oxidoreductase, which is responsible for sorbitol production, showed the minimum and the maximum fluxes of 0 and 20 mmol gDW^−1^.h^−1^, respectively. According to experimental evidence, sorbitol and levan are the products of sucrose utilization^33^. Therefore, our model could show that these pathways are practical in the presence of sucrose and fructose as carbon sources.

### Model evaluation based on genetic engineering data

#### Xylose utilization

Glucose and xylose are the main derivatives of lignocellulosic biomass digestion. Therefore, the conversion of these carbon sources to ethanol is economically important. *Z. mobilis* lacks a complete pentose utilization pathway. Therefore, many efforts have been taken to introduce the necessary genes to the metabolism of this microorganism^1^. As shown in Table 2, xylose isomerase, xylulokinase, transketolase and transaldolase activity is necessary to provide Entner-Doudoroff pathway the intermediates required for converting xylose to ethanol^34^. We simulated the growth of *Z. mobilis* on minimal medium, and additionally, permitted xylose to go into the system by setting the lower bound of its exchange reaction to −10 mmol gDW^−1^ h^−1^. Investigation of the modeling predictions for growth rate in this case by running FBA showed 0.136 mmol gDW^−1^.h^−1^. The *in silico* ethanol production simulations were done using FBA. In the case of glucose and xylose consumption, 29.42 and 21.92 mmol gDW^−1^.h^−1^ ethanol was produced, respectively. In the mixed culture of glucose and xylose, 49.42 mmol gDW^−1^.h^−1^ ethanol production was obtained. The results showed that ethanol production would be improved in mixed culture as more carbon sources are available for the microorganism. Based on growth and ethanol production, the modeling outcome is compatible with experimental results.

**Table 2:** Results of the comprehensive literature search for genetic manipulation of *Z. mobilis*.

Expanding carbon sources		
Engineering purpose	Heterologous Enzymes	Reference
Xylose utilization	xylose isomerase, xylulokinase, transaldolase	Zhang and Eddy, 1995
Arabinose utilization	ribulokinase, arabinose isomerase, ribulose-5-phosphate-4-epimerase, transaldolase	Deanda *et al*., 1996
**Expanding products**		
Alanine production	Alanine dehydrogenase	Uhlenbusch *et al*., 1991
β-carotene production	geranylgeranyl diphosphate synthase, phytoene synthase, phytoene desaturase, lycopene cyclase	Misawa *et al*., 1991
2,3-butanediol production	butanediol dehydrogenase	Yang *et al*., 2016b
**Knock-out of genes**		
Omitting sorbitol production by *gfo* knock-out	Glucose-fructose oxidoreductase (*gfo*)	Wang *et al*., 2013
succinic acid overproduction	pyruvate decarboxylase (*pdc*) and lactate dehydrogenase (*ldh*)	Rogers *et al*., 2007

#### Arabinose utilization

Along with the previous attempts for expanding substrate usage ability in *Z. mobilis*, arabinose utilization genes were inserted into this bacterium. Similar to the case of xylose, arabinose is another five-carbon sugar that is found in lignocellulosic biomass. Arabinose isomerase, ribulose-5-phosphate-4-epimerase, ribulokinase and transaldolase were added to the model^35^. The lower bound was set to −10 mmol gDW^−1^ h^−1^ in arabinose exchange reaction. By performing FBA, the model predicted 0.136 mmol gDW^−1^ h^−1^ for the growth rate and 24.42 mmol gDW^−1^ h^−1^ ethanol production. Therefore, the simulations reflected the experimental observations adequately.

#### Alanine production

Alanine dehydrogenase gene has been previously employed to demonstrate successive improvements in broadening the product formation in *Z. mobilis*^36^. By engineering this pathway, flux from ethanol production is redirected to alanine production. In order to analyze the splitting of carbon sources to these two end products, we used FBA. Our modeling observation is compatible with experimental results and showed less ethanol, 28.45 mmol gDW^−1^ h^−1^ (compared to 29.42) when alanine is produced.

#### β- carotene production

β-carotene is popular mainly because of its vast applications in the production of pharmaceuticals and as a food additive. Biosynthesis of β-carotene is achieved by heterologous expression of β-carotene synthesizing genes in an appropriate host like *Z. mobilis*^37^. As presented in Table 2, geranylgeranyl diphosphate synthase, phytoene desaturase, phytoene synthase and lycopene cyclase were added into the metabolic model. The model was then evaluated to examine the activity of the aforementioned pathway. For simulation tests, the upper bounds and lower bounds of the corresponding reactions were set to +1000 mmol gDCW^−1^ h^−1^ and −1000 mmol gDCW^−1^ h^−1^, respectively. The β-carotene production reaction flux was predicted to be 1.016 mmol gDW-1.h-1. Therefore, our results of FBA demonstrated that the aforementioned pathway is active when β-carotene synthesizing genes were provided.

#### 2,3-butanediol production

Microbial production of 2,3-butanediol, which is often produced petrochemically, has gained scientists’ attention in recent years. Production of this important chemical may be achieved by introducing related genes to *Z. mobilis*^38^. Here we added butanediol dehydrogenase gene to *Z. mobilis* metabolic network to model the engineered strain. In order to study the modeling predictions in this condition, we kept the glucose and/or xylose uptake rate at −10 mmol gDW^−1^ h^−1^ and allowed butanediol dehydrogenase activity by adding the constraint −1000 mmol gDCW^−1^ h^−1^ and 1000 mmol gDCW^−1^ h^−1^ to see how it would affect the butanediol production. By using FBA, in the case of glucose or xylose consumption, 14.71 and 10.96 mmol gDCW^−1^ h^−1^ was produced, respectively. As butanediol production was 24.71 mmol gDCW^−1^ h^−1^, the model could reflect the experimental observations of butanediol production in the mixed culture, successfully,

#### gfo gene knockout

The glucose-fructose oxidoreductase is encoded by *gfo* gene. In a previous study, a *gfo* knockout in *Z. mobilis* was constructed and the metabolic features of this bacterium were studied^33^. Here, we report the consequences of the experimental and *in silico* simulation of these evaluates. In experimental results, the inactivation of *gfo* had different results on the growth of *Z. mobilis* at high or low concentration of glucose, fructose or sucrose. However, as shown in the simulation results, the model could not predict the experimental observations correctly. These observations proposed that these effects may be related to regulation mechanisms in *Z. mobilis*. So, more research is needed in differences in transcription profile in these conditions.

#### pdc and ldh gene knockout

Succinic acid can be used in numerous applications like surfactants, detergents and solvents or food and pharmaceutical products. *pdc* and *ldh* are genes that participate in lactate and ethanol production. By omitting these two genes, fluxes are redirected to succinic acid production and resulted in improved concentrations of succinic acid^39^. By running FVA, when ethanol and lactate were produced, minimum and maximum fluxes of succinic acid production were 0 and 0.33 mmol gDCW^−1^ h^−1^, respectively. Here we knocked out the above two genes *in silico*, so maximum succinic acid production changed to 5.33 mmol gDCW^−1^ h^−1^. Consequently, more succinic acid production in knock-out condition was confirmed.

### Model evaluation based on gene essentiality analysis

A recently-published article reports that, floc formation in a defined minimal medium under the aerobic condition is essential for *Z. mobilis* ZM4 survival. In this case, a gene cluster of cellulose synthase was found to be vital for microorganism^40^. It should be noticed that, cells within flocs are linked together by a cellulosic extracellular matrix. So, we added a new cellulose-containing biomass reaction to be used for modeling in minimal medium. By simulating this minimal medium in aerobic condition, our model could reflect the essentiality of cellulose synthase, successfully.

### Model evaluation based on transcriptomic data

#### Evaluation based on Reporter Metabolite Algorithm

**Figure 5:**
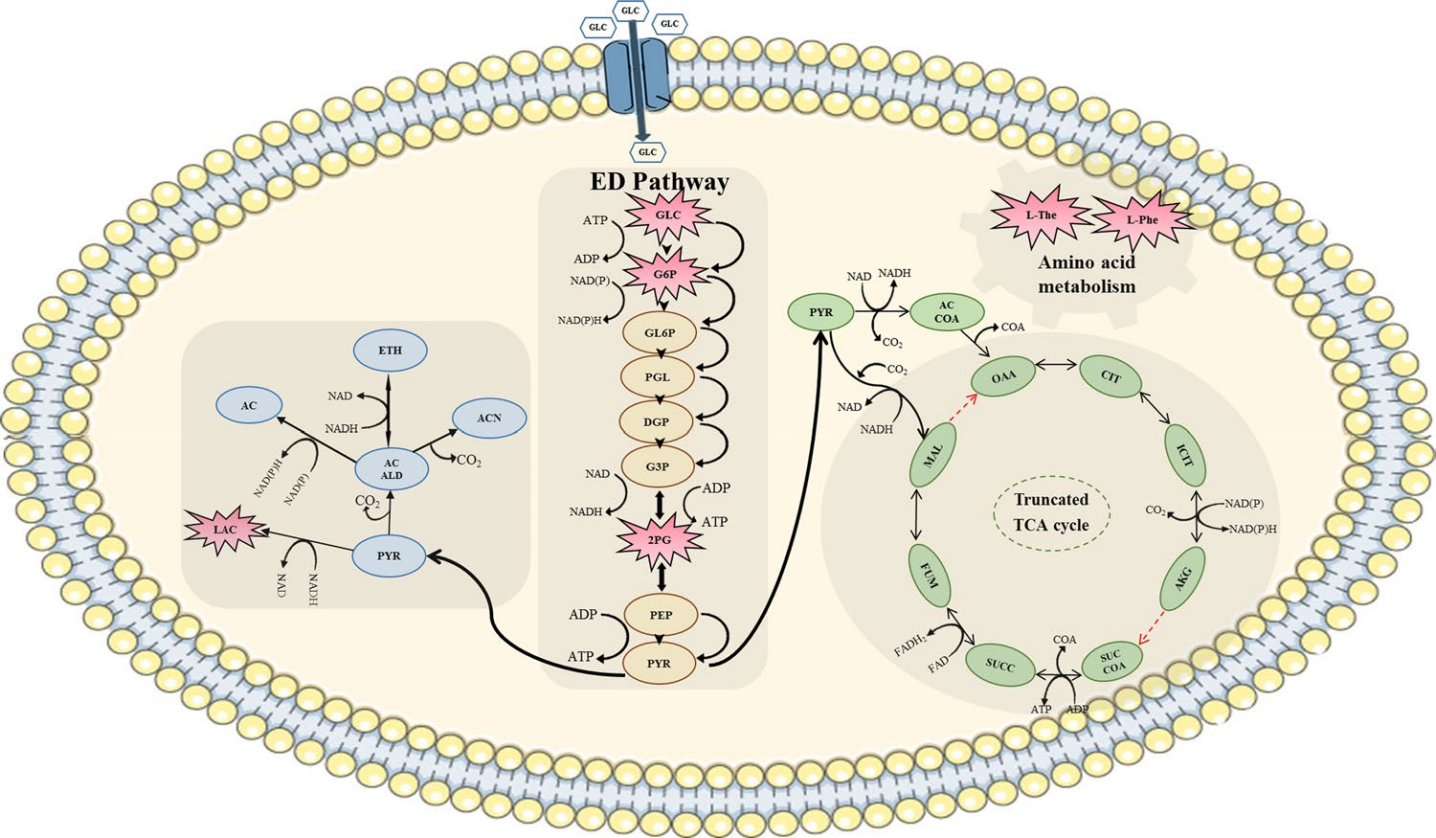
Confirmed metabolites in different pathways of *Z. mobilis* based on reporter metabolite algorithm results that are linked to the transcriptional changes during aerobic and anaerobic fermentation. Cell membrane image was obtained from (https://smart.servier.com/) CC BY 3.0.

As a qualitative validation of predicted flux distributions by a GEM, one can take advantage of transcriptomic data. For *Z. mobilis* ZM4, the maximum specific growth rates are comparable under aerobic and anaerobic conditions, while genes related to ethanol (and other byproducts) formation may be differentially regulated, which results in differences in the transcriptomic profiles^26^. For validating gene activity of different pathways under aerobic *vs*. anaerobic conditions, in the first step, the “reporter metabolite algorithm” was used^41^. In this method, by integrating transcriptome data with a GEM, key metabolites that are linked to the most significant transcriptional changes are identified. Glucose 6-phosphate, glycerate, glucose, 2-Phosphoglycerate, l-Phenylalanine, l-Threonine and lactate were found different in the metabolomic profile of *Z. mobilis* during aerobic and anaerobic fermentation based on reporter metabolite algorithm results (Fig. 5). These findings confirmed the physiological status of *Z. mobilis* that was investigated by GC-MS analysis^26^.

As it was indicated in previous research, metabolism of sulfur compounds is impacted under these two conditions^26^. Thiosulfate, sulfo-l-cysteine, l-Selenocysteine, 3-Sulfino-l-alanine are sulfur-containing metabolites for which a statistically significant difference in response to environmental aeration condition was observed. Although 4-hydroxybutanoate that is differentially produced between the above conditions is not present in our metabolic network, 2-Hydroxy-2-methyl-3-oxobutanoate, 2-Aceto-2-hydroxybutanoate and 2,3-Dihydroxy-3-methylbutanoate that are butanoate-derivatives are shown to be related to aerated fermentation. Overall, using an updated GEM, we elucidated how changes in metabolic state occur during anaerobic *vs*. aerobic shift conditions.

#### Evaluation based on uniform random sampling of flux space

We compared flux changes between aerobic and anaerobic conditions by using a random sampling of the metabolic flux space^42^. By comparing the distribution of that flux value under aerobic *vs*. anaerobic conditions, each results with *P*-values lower than 0.05 considered statistically significant. Also, previously published transcript data in response to anaerobiosis introduced up or down-regulated genes. Then, the list of significantly increased (or decreased) reaction fluxes were compared with the list of up- (or down-) regulated genes based on the transcriptomic data. Comparison of transcriptomes with flux data showed, while some of these changes were consistent with previous transcriptome studies, most of them are not metabolic genes. These observations are the evidence of the role of transcriptionally controlled mechanisms and showed new insights into the physiology of anaerobically growing *Z. mobilis* ZM4.

Our results demonstrated that pathways most significantly impacted by aeration shift were glycolysis, amino acid, purine and pyrimidine metabolism. Between reactions pyruvate decarboxylase and alcohol dehydrogenase had significantly lower flux in aerobic condition. This finding is in accordance with experimental evidence. We also evaluate our results with up or down-regulated genes that were obtained from transcriptomic data. Additionally, we assessed the accordance between results achieved from reporter metabolite algorithm with sampling results. In the case of up-regulated genes, 36% of the sampling positive results were confirmed by the transcriptomic data, while the down-regulated genes were 73% consistent with sampling results. While our results showed an acceptable level of correctness (accuracy = 0.67, F1 score = 0.77), but there is still room for improvement. (Table 3).

**Table 3:** Comparison of transcriptomic experimental observations and computational random sampling results of up or down-regulated metabolic genes in aerobic *vs*. anaerobic condition.

Down-regulated metabolic genes/consistent		Up-regulated metabolic genes/consistent	
ZMO0105	3-isopropylmalate dehydratase large subunit	ZMO0311	Pyrroline-5-carboxylate reductase
ZMO0585	Tryptophan synthase beta chain	ZMO1617	Carbamoyl-phosphate synthase large chain
ZMO0804	N-acetyl-gamma-glutamyl-phosphate reductase	ZMO1460	Thiosulfate sulfurtransferase
ZMO1139	Acetolactate synthase large subunit	ZMO1286	Sorbitol dehydrogenase small subunit
ZMO1141	Ketol-acid reductoisomerase		
ZMO1407	Aspartate-semialdehyde dehydrogenase		
ZMO1891	Threonine synthase		
ZMO1321	Inosine-5-monophosphate dehydrogenase		
ZMO0475	Riboflavin synthase alpha chain		
ZMO1571	Cytochrome bd-type quinol oxidase subunit 1		
ZMO1572	Cytochrome bd-type quinol oxidase subunit 2		
ZMO1113	NADH dehydrogenase		
ZMO0152	Pyruvate kinase		
ZMO0369	Glucokinase		
ZMO1240	Phosphoglycerate mutase		
ZMO1596	Alcohol dehydrogenase II		
ZMO1608	Enolase		
ZMO1649	Gluconolactonase		
ZMO1719	Fructokinase		
**Down-regulated metabolic genes/inconsistent**		**Up-regulated metabolic genes/inconsistent**	
ZMO0172	Thiamine biosynthesis protein	ZMO1853	Dihydrodipicolinate synthase
ZMO0889	Aldose 1-epimerase precursor	ZMO1792	Dihydroxy-acid dehydratase
ZMO0239	ATP synthase alpha subunit	ZMO1887	Isochorismatase
ZMO0241	ATP synthase beta subunit	ZMO1879	Delta-aminolevulinic acid dehydratase
ZMO0367	Glucose-6-phosphate dehydrogenase	ZMO1489	3-deoxy-D-manno-octulosonate cytidylyltransferase
ZMO0465	Triosephosphate isomerase	ZMO1496	Phosphoenolpyruvate carboxylase
ZMO1478	6-phosphogluconolactonase	ZMO1347	Threonine aldolase

## Conclusion

In this work, an upgraded high-quality metabolic model of *Z. mobilis* ZM4, *i*HN446, is presented. The model is more comprehensive in scope than previous models and updated based on the latest gene annotations, databases and literature. The model validation was achieved by comparing model predictions to experimentally gained results or from the literature. *i*HN446 showed desirable simulated performance when predicting specific growth rate on the carbon sources and the effects of gene deletion or gene insertion on cell growth and/or product formation. We also investigated the predictive power of the model based on transcription data in aerobic and anaerobic conditions by comparing changes in the flux of each reaction *vs*. transcription data. This advanced and comprehensive genome-scale metabolic model, make a potential platform for a better understanding of *Z. mobilis* metabolic features at a systems-level and is a valuable tool to develop engineered *Z. mobilis* strains for several biotechnological applications. Finally, this model can be a framework for the integration of transcriptomic data to provide more accurate metabolic engineering predictions.

## Supplementary information

Supplementary Dataset 1.

Supplementary Dataset 2.

Supplementary Table S3.

Supplementary Table S4.

## Data Availability

All data generated or analyzed during this study are included in this published article (and its Supplementary Information Files).
