# Supplementary material for: A reconciliation of genome-scale metabolic network model of Zymomonas mobilis ZM4
Source: Sci Rep. 2020 May 8;10:7782. doi: 10.1038/s41598-020-64721-x (PMC7210963; doi:10.1038/s41598-020-64721-x)
Supplement: Supplementary file 3 — Supplementary Table S3. [file 41598_2020_64721_MOESM3_ESM.docx]

**A reconciliation of genome-scale metabolic network model of *Zymomonas mobilis* ZM4**

Hoda Nouri^1^, Hamideh Fouladiha^2^, Hamid Moghimi^*1^, Sayed-Amir Marashi^*2^

**S3 Table 1. Enzymes comparison and Genes comparison**

S3 Table 1. Enzymes comparison

| **Enzymes comparison** | | | |
| --- | --- | --- | --- |
| **ZmoMBEL601** | ***i*HN446** | ***i*ZM363** | ***i*HN446** |
| 1.1.1.158 | 1.1.1.14 | 1.1.1.158 | 1.1.1.100 |
| 1.17.1.2 | 1.1.1.264 | 1.17.1.2 | 1.1.1.25 |
| 1.2.1.3 | 1.1.1.274 | 1.3.1.26 | 1.1.1.276 |
| 1.3.1.26 | 1.1.1.290 | 1.3.3.1 | 1.1.1.304 |
| 1.3.3.1 | 1.1.1.69 | 1.3.99.1 | 1.1.1.343 |
| 1.3.99.1 | 1.2.1.21 | 2.1.1.- | 1.1.1.346 |
| 2.1.1.- | 1.2.1.72 | 2.3.1.41 | 1.1.1.388 |
| 2.3.1.41 | 1.3.1.12 | 2.5.1.- | 1.1.1.399 |
| 2.5.1.- | 1.3.1.9 | 2.7.8.- | 1.1.1.405 |
| 2.7.8.- | 1.3.5.1 | 4.2.1.52 | 1.1.1.67 |
| 3.1.1.- | 2.1.1.71 | 5.4.2.1 | 1.1.1.363 |
| 4.1.1.- | 2.3.1.38 | 1.1.1.282 | 1.1.1.76 |
| 4.1.1.74 | 2.5.1.48 | 1.1.1.35 | 1.1.1.78 |
| 4.2.-.- | 2.5.1.55 | 1.1.1.47 | 1.1.1.79 |
| 4.2.1.52 | 2.6.1.13 | 1.1.1.9 | 1.1.1.81 |
| 5.4.2.1 | 2.6.1.81 | 1.10.2.- | 1.1.5.12 |
|  | 2.7.1.20 | 1.13.11.15 | 1.1.99.21 |
|  | 2.7.1.48 | 1.13.11.27 | 1.1.99.28 |
|  | 2.7.1.49 | 1.14.13.18 | 1.1.99.3 |
|  | 2.7.1.50 | 1.14.13.3 | 1.10.3.14 |
|  | 2.7.7.38 | 1.14.3.- | 1.11.1.5 |
|  | 3.6.1.23 | 1.15.1.1 | 1.13.11.54 |
|  | 4.1.1.47 | 1.2.1.10 | 1.14.13.- |
|  | 5.1.2.2 | 1.2.1.18 | 1.14.13.82 |
|  | 5.3.1.13 | 1.2.1.28 | 1.14.99.60 |
|  | 5.4.3.8 | 1.2.1.60 | 1.16.3.1 |
|  | 5.4.4.2 | 1.2.1.7 | 1.17.1.8 |
|  | 6.1.1.1 | 1.2.7.1 | 1.17.7.3 |
|  | 6.1.1.10 | 1.3.99.2 | 1.17.7.4 |
|  | 6.1.1.11 | 1.3.99.3 | 1.17.8.1 |
|  | 6.1.1.12 | 1.5.1.15 | 1.18.1.2 |
|  | 6.1.1.14 | 1.6.5.3 | 1.18.6.1 |
|  | 6.1.1.15 | 1.8.2.2 | 1.2.1.- |
|  | 6.1.1.16 | 2.1.2.- | 1.2.1.20 |
|  | 6.1.1.17 | 2.2.1.2 | 1.2.1.5 |
|  | 6.1.1.19 | 2.3.1.29 | 1.2.1.70 |
|  | 6.1.1.2 | 2.3.1.9 | 1.2.1.79 |
|  | 6.1.1.20 | 2.3.3.9 | 1.3.1.10 |
|  | 6.1.1.21 | 2.4.1.18 | 1.3.1.78 |
|  | 6.1.1.3 | 2.4.2.- | 1.3.1.79 |
|  | 6.1.1.4 | 2.4.2.8 | 1.3.1.98 |
|  | 6.1.1.5 | 2.6.1.19 | 1.3.3.4 |
|  | 6.1.1.6 | 2.6.1.5 | 1.3.98.1 |
|  | 6.1.1.7 | 2.6.1.58 | 1.3.99.22 |
|  | 6.1.1.9 | 2.7.1.16 | 1.4.1.14 |
|  | 6.3.5.4 | 2.7.1.17 | 1.4.1.3 |
|  | 1.1.1.100 | 2.7.1.30 | 1.4.4.2 |
|  | 1.1.1.276 | 2.7.1.66 | 1.5.1.1 |
|  | 1.1.1.304 | 2.7.6.1 | 1.6.5.2 |
|  | 1.1.1.343 | 2.8.1.1 | 1.6.99.3 |
|  | 1.1.1.346 | 3.2.2.2 | 1.7.1.13 |
|  | 1.1.1.388 | 3.5.1.16 | 1.7.1.4 |
|  | 1.1.1.399 | 3.5.2.5 | 1.7.7.2 |
|  | 1.1.1.405 | 3.5.4.1 | 1.7.99.1 |
|  | 1.1.1.67 | 3.6.1.- | 1.8.1.2 |
|  | 1.1.1.363 | 3.6.1.41 | 1.8.4.10 |
|  | 1.1.1.76 | 4.1.1.21 | 2.1.1.16 |
|  | 1.1.1.78 | 4.1.1.49 | 2.1.1.163 |
|  | 1.1.5.12 | 4.1.1.7 | 2.1.1.197 |
|  | 1.1.99.28 | 4.1.1.72 | 2.1.1.201 |
|  | 1.1.99.3 | 4.1.2.- | 2.1.1.222 |
|  | 1.10.3.14 | 4.1.2.4 | 2.1.2.10 |
|  | 1.11.1.5 | 4.1.3.3 | 2.1.2.9 |
|  | 1.13.11.54 | 4.1.3.7 | 2.1.3.15 |
|  | 1.14.99.60 | 4.2.1.- | 2.3.1.179 |
|  | 1.16.3.1 | 4.2.1.55 | 2.3.1.180 |
|  | 1.17.1.8 | 4.2.1.60 | 2.3.1.181 |
|  | 1.17.7.3 | 5.1.2.3 | 2.3.1.37 |
|  | 1.17.7.4 | 5.1.3.2 | 2.3.1.79 |
|  | 1.17.8.1 | 5.1.3.4 | 2.3.3.14 |
|  | 1.2.1.- | 5.3.1.4 | 2.4.1.1 |
|  | 1.2.1.20 | 5.3.1.5 | 2.4.1.10 |
|  | 1.2.1.5 | 5.3.3.10 | 2.4.1.80 |
|  | 1.2.1.70 | 5.4.2.7 | 2.4.1.83 |
|  | 1.2.1.79 | 6.2.1.3 | 2.4.2.15 |
|  | 1.3.1.10 | 6.3.2.15 | 2.4.2.2 |
|  | 1.3.1.78 | 6.3.4.1 | 2.4.2.3 |
|  | 1.3.1.79 | 6.3.5.1 | 2.4.2.4 |
|  | 1.3.1.98 | 6.3.5.8 | 2.4.2.7 |
|  | 1.3.3.4 | 1.1.1.158 | 2.4.2.7 |
|  | 1.3.98.1 | 1.17.1.2 | 2.5.1.1 |
|  | 1.4.4.2 | 1.3.1.26 | 2.5.1.103 |
|  | 1.5.1.1 | 1.3.3.1 | 2.5.1.129 |
|  | 1.6.5.2 | 1.3.99.1 | 2.5.1.17 |
|  | 1.6.99.3 | 2.1.1.- | 2.5.1.18 |
|  | 1.7.1.13 | 2.3.1.41 | 2.5.1.21 |
|  | 1.7.1.4 | 2.5.1.- | 2.5.1.31 |
|  | 1.7.7.2 | 2.7.8.- | 2.5.1.39 |
|  | 1.7.99.1 | 4.2.1.52 | 2.5.1.78 |
|  | 1.8.4.10 | 5.4.2.1 | 2.5.1.90 |
|  | 2.1.1.16 | 1.1.1.282 | 2.6.1.2 |
|  | 2.1.1.163 | 1.1.1.35 | 2.6.1.85 |
|  | 2.1.1.197 | 1.1.1.47 | 2.7.1.170 |
|  | 2.1.1.201 | 1.1.1.9 | 2.7.1.8 |
|  | 2.1.1.222 | 1.10.2.- | 2.7.4.22 |
|  | 2.1.2.10 | 1.13.11.15 | 2.7.4.25 |
|  | 2.1.2.9 | 1.13.11.27 | 2.7.6.1 |
|  | 2.1.3.15 | 1.14.13.18 | 2.7.6.5 |
|  | 2.3.1.179 | 1.14.13.3 | 2.7.7.1 |
|  | 2.3.1.180 | 1.14.3.- | 2.7.7.13 |
|  | 2.3.1.181 | 1.15.1.1 | 2.7.7.40 |
|  | 2.3.1.79 | 1.2.1.10 | 2.7.7.99 |
|  | 2.4.1.83 | 1.2.1.18 | 2.7.8.41 |
|  | 2.4.2.15 | 1.2.1.28 | 2.8.1.1 |
|  | 2.4.2.2 | 1.2.1.60 | 2.8.1.10 |
|  | 2.4.2.3 | 1.2.1.7 | 2.8.1.2 |
|  | 2.4.2.4 | 1.2.7.1 | 2.8.1.8 |
|  | 2.4.2.7 | 1.3.99.2 | 3.1.1.24 |
|  | 2.5.1.1 | 1.3.99.3 | 3.1.1.92 |
|  | 2.5.1.103 | 1.5.1.15 | 3.1.2.12 |
|  | 2.5.1.129 | 1.6.5.3 | 3.1.3.- |
|  | 2.5.1.17 | 1.8.2.2 | 3.1.3.102 |
|  | 2.5.1.39 | 2.1.2.- | 3.1.3.26 |
|  | 2.5.1.78 | 2.2.1.2 | 3.1.3.37 |
|  | 2.5.1.90 | 2.3.1.29 | 3.1.3.45 |
|  | 2.7.1.170 | 2.3.1.9 | 3.1.3.77 |
|  | 2.7.1.8 | 2.3.3.9 | 3.2.1.23 |
|  | 2.7.4.22 | 2.4.1.18 | 3.2.1.52 |
|  | 2.7.4.25 | 2.4.2.- | 3.2.2.14 |
|  | 2.7.6.5 | 2.4.2.8 | 3.4.11.1 |
|  | 2.7.7.13 | 2.6.1.19 | 3.4.19.13 |
|  | 2.7.7.40 | 2.6.1.5 | 3.5.1.42 |
|  | 2.7.7.99 | 2.6.1.58 | 3.5.1.53 |
|  | 2.7.8.41 | 2.7.1.16 | 3.5.1.68 |
|  | 2.8.1.1 | 2.7.1.17 | 3.5.1.96 |
|  | 2.8.1.10 | 2.7.1.30 | 3.5.3.12 |
|  | 2.8.1.2 | 2.7.1.66 | 3.5.4.2 |
|  | 2.8.1.8 | 2.7.6.1 | 3.5.5.1 |
|  | 3.1.1.92 | 2.8.1.1 | 3.6.1.11 |
|  | 3.1.2.12 | 3.2.2.2 | 3.6.1.13 |
|  | 3.1.3.102 | 3.5.1.16 | 3.6.1.15 |
|  | 3.1.3.37 | 3.5.2.5 | 3.6.1.19 |
|  | 3.1.3.45 | 3.5.4.1 | 3.6.1.22 |
|  | 3.2.2.14 | 3.6.1.- | 3.6.1.40 |
|  | 3.4.19.13 | 3.6.1.41 | 3.6.1.66 |
|  | 3.5.1.42 | 4.1.1.21 | 3.6.1.8 |
|  | 3.5.1.96 | 4.1.1.49 | 4.1.1.11 |
|  | 3.5.4.2 | 4.1.1.7 | 4.1.1.12 |
|  | 3.6.1.22 | 4.1.1.72 | 4.1.1.98 |
|  | 3.6.1.40 | 4.1.2.- | 4.1.2.50 |
|  | 3.6.1.66 | 4.1.2.4 | 4.1.3.- |
|  | 3.6.1.8 | 4.1.3.3 | 4.1.3.16 |
|  | 4.1.1.98 | 4.1.3.7 | 4.1.3.40 |
|  | 4.1.2.50 | 4.2.1.- | 4.1.99.12 |
|  | 4.1.3.40 | 4.2.1.55 | 4.1.99.17 |
|  | 4.1.99.17 | 4.2.1.60 | 4.2.1.109 |
|  | 4.2.1.126 | 5.1.2.3 | 4.2.1.126 |
|  | 4.2.1.129 | 5.1.3.2 | 4.2.1.129 |
|  | 4.2.1.153 | 5.1.3.4 | 4.2.1.153 |
|  | 4.2.1.59 | 5.3.1.4 | 4.2.1.35 |
|  | 4.2.3.156 | 5.3.1.5 | 4.2.1.59 |
|  | 4.3.3.7 | 5.3.3.10 | 4.2.3.12 |
|  | 4.3.99.3 | 5.4.2.7 | 4.2.3.156 |
|  | 4.4.1.22 | 6.2.1.3 | 4.3.1.12 |
|  | 5.1.1.20 | 6.3.2.15 | 4.3.3.7 |
|  | 5.1.3.6 | 6.3.4.1 | 4.3.99.3 |
|  | 5.3.1.22 | 6.3.5.1 | 4.4.1.11 |
|  | 5.4.2.11 | 6.3.5.8 | 4.4.1.22 |
|  | 6.1.1.18 | 4.1.2.4 | 5.1.1.13 |
|  | 6.3.4.20 | 4.1.3.3 | 5.1.1.20 |
|  | 6.3.4.21 | 4.1.3.7 | 5.1.3.12 |
|  | 6.3.5.6 | 4.2.1.- | 5.1.3.3 |
|  | 6.6.1.2 | 4.2.1.55 | 5.1.3.6 |
|  | 4.2.1.153 | 4.2.1.60 | 5.3.1.22 |
|  | 4.2.1.59 | 5.1.2.3 | 5.3.3.2 |
|  | 4.2.3.156 | 5.1.3.2 | 5.4.2.11 |
|  | 4.3.3.7 | 5.1.3.4 | 5.4.99.17 |
|  | 4.3.99.3 | 5.3.1.4 | 5.5.1.4 |
|  | 4.4.1.22 | 5.3.1.5 | 6.1.1.18 |
|  | 5.1.1.20 | 5.3.3.10 | 6.3.1.1 |
|  | 5.1.3.6 | 5.4.2.7 | 6.3.2.17 |
|  | 5.3.1.22 | 6.2.1.3 | 6.3.4.20 |
|  | 5.4.2.11 | 6.3.2.15 | 6.3.4.21 |
|  | 6.1.1.18 | 6.3.4.1 | 6.3.5.6 |
|  | 6.3.4.20 | 6.3.5.1 | 6.6.1.2 |
|  | 6.3.4.21 | 6.3.5.8 | 3.1.4.11 |
|  | 6.3.5.6 | 5.3.3.10 | 2.6.1.37 |
|  | 6.6.1.2 | 5.4.2.7 | 4.1.1.44 |
|  | 5.1.3.6 | 6.2.1.3 | 3.5.1.28 |
|  | 5.3.1.22 | 6.3.2.15 | 2.4.1.12 |
|  | 5.4.2.11 | 6.3.4.1 |  |
|  | 6.1.1.18 | 6.3.5.1 |  |
|  | 6.3.4.20 | 6.3.5.8 |  |
|  | 6.3.4.21 |  |  |
|  | 6.3.5.6 |  |  |
|  | 6.6.1.2 |  |  |
|  | 3.1.4.11 |  |  |
|  | 2.6.1.37 |  |  |
|  | 4.1.1.44 |  |  |
|  | 3.5.1.28 |  |  |
|  | 2.4.1.12 |  |  |

*Differences between ZmoMBEL601/*i*HN446 and *i*ZM363/*i*HN446 in enzymes

S3 Table 1. Genes comparison

| **Genes comparison** | | | |
| --- | --- | --- | --- |
| ***i*HN446** | **ZmoMBEL601** | ***i*HN446** | ***i*ZM363** |
| ZMO0115 | ZMO0120 | ZMO0115 | ZMO0017 |
| ZMO0117 | ZMO0736 | ZMO0117 | ZMO0099 |
| ZMO0226 | ZMO0893 | ZMO0226 | ZMO0168 |
| ZMO0318 |  | ZMO0318 | ZMO0232 |
| ZMO0326 |  | ZMO0326 | ZMO0402 |
| ZMO0343 |  | ZMO0343 | ZMO0570 |
| ZMO0353 |  | ZMO0353 | ZMO0574 |
| ZMO0366 |  | ZMO0366 | ZMO0761 |
| ZMO0446 |  | ZMO0446 | ZMO0765 |
| ZMO0486 |  | ZMO0486 | ZMO0823 |
| ZMO0503 |  | ZMO0503 | ZMO0845 |
| ZMO0558 |  | ZMO0558 | ZMO0865 |
| ZMO0689 |  | ZMO0689 | ZMO0952 |
| ZMO0713 |  | ZMO0713 | ZMO0957 |
| ZMO0734 |  | ZMO0734 | ZMO0958 |
| ZMO0738 |  | ZMO0738 | ZMO1032 |
| ZMO0782 |  | ZMO0782 | ZMO1060 |
| ZMO0783 |  | ZMO0783 | ZMO1179 |
| ZMO0784 |  | ZMO0784 | ZMO1203 |
| ZMO0811 |  | ZMO0811 | ZMO1314 |
| ZMO0869 |  | ZMO0869 | ZMO1344 |
| ZMO0870 |  | ZMO0870 | ZMO1417 |
| ZMO0871 |  | ZMO0871 | ZMO1435 |
| ZMO0872 |  | ZMO0872 | ZMO1502 |
| ZMO1023 |  | ZMO1023 | ZMO1554 |
| ZMO1100 |  | ZMO1100 | ZMO1562 |
| ZMO1113 |  | ZMO1113 | ZMO1640 |
| ZMO1130 |  | ZMO1130 | ZMO1643 |
| ZMO1132 |  | ZMO1132 | ZMO1644 |
| ZMO1136 |  | ZMO1136 | ZMO1646 |
| ZMO1154 |  | ZMO1154 | ZMO1837 |
| ZMO1169 |  | ZMO1169 | ZMO1889 |
| ZMO1222 |  | ZMO1222 | ZMO1921 |
| ZMO1284 |  | ZMO1284 | ZMO0017 |
| ZMO1286 |  | ZMO1286 | ZMO0099 |
| ZMO1302 |  | ZMO1302 | ZMO0168 |
| ZMO1309 |  | ZMO1309 | ZMO0232 |
| ZMO1335 |  | ZMO1335 | ZMO0402 |
| ZMO1410 |  | ZMO1410 | ZMO0570 |
| ZMO1449 |  | ZMO1449 | ZMO0574 |
| ZMO1495 |  | ZMO1495 | ZMO0761 |
| ZMO1530 |  | ZMO1530 | ZMO0765 |
| ZMO1543 |  | ZMO1543 | ZMO0823 |
| ZMO1544 |  | ZMO1544 | ZMO0845 |
| ZMO1548 |  | ZMO1548 | ZMO0865 |
| ZMO1571 |  | ZMO1571 | ZMO0952 |
| ZMO1572 |  | ZMO1572 | ZMO0957 |
| ZMO1586 |  | ZMO1586 | ZMO0958 |
| ZMO1642 |  | ZMO1642 | ZMO1032 |
| ZMO1652 |  | ZMO1652 | ZMO1060 |
| ZMO1806 |  | ZMO1806 | ZMO1179 |
| ZMO1899 |  | ZMO1899 | ZMO1203 |
| ZMO1900 |  | ZMO1900 | ZMO1314 |
| ZMO1946 |  | ZMO1946 | ZMO1344 |
| ZMO1949 |  | ZMO1949 | ZMO1417 |
| ZMO0186 |  | ZMO0008 | ZMO1435 |
| ZMO0239 |  | ZMO0009 | ZMO1502 |
| ZMO0240 |  | ZMO0012 | ZMO1554 |
| ZMO0241 |  | ZMO0013 | ZMO1562 |
| ZMO0242 |  | ZMO0041 | ZMO1640 |
| ZMO0323 |  | ZMO0053 | ZMO1643 |
| ZMO0460 |  | ZMO0113 | ZMO1644 |
| ZMO0637 |  | ZMO0114 | ZMO1646 |
| ZMO0667 |  | ZMO0172 | ZMO1837 |
| ZMO0668 |  | ZMO0371 | ZMO1889 |
| ZMO0669 |  | ZMO0374 | ZMO1921 |
| ZMO0671 |  | ZMO0403 |  |
| ZMO0715 |  | ZMO0421 |  |
| ZMO0843 |  | ZMO0456 |  |
| ZMO0890 |  | ZMO0560 |  |
| ZMO0900 |  | ZMO0562 |  |
| ZMO0986 |  | ZMO0676 |  |
| ZMO1092 |  | ZMO0788 |  |
| ZMO1146 |  | ZMO0806 |  |
| ZMO1191 |  | ZMO0818 |  |
| ZMO1228 |  | ZMO0889 |  |
| ZMO1264 |  | ZMO0904 |  |
| ZMO1303 |  | ZMO0935 |  |
| ZMO1444 |  | [ZMO0941](http://www.genome.jp/dbget-bin/www_bget?zmo:ZMO0941) |  |
| ZMO1446 |  | ZMO0972 |  |
| ZMO1460 |  | ZMO1041 |  |
| ZMO1488 |  | ZMO1059 |  |
| ZMO1489 |  | ZMO1095 |  |
| ZMO1513 |  | ZMO1118 |  |
| ZMO1514 |  | ZMO1152 |  |
| ZMO1531 |  | ZMO1158 |  |
| ZMO1532 |  | ZMO1171 |  |
| ZMO1606 |  | ZMO1181 |  |
| ZMO1692 |  | ZMO1189 |  |
| ZMO1878 |  | ZMO1195 |  |
| ZMO1964 |  | ZMO1198 |  |
| ZMO1083 |  | ZMO1207 |  |
| ZMOp32x007 |  | ZMO1278 |  |
| ZMOp33x012 |  | ZMO1294 |  |
| ZMOp33x017 |  | ZMO1360 |  |
| ZMOp36x001 |  | ZMO1364 |  |
|  |  | ZMO1369 |  |
|  |  | ZMO1370 |  |
|  |  | ZMO1425 |  |
|  |  | ZMO1500 |  |
|  |  | ZMO1669 |  |
|  |  | ZMO1682 |  |
|  |  | ZMO1686 |  |
|  |  | ZMO1703 |  |
|  |  | ZMO1753 |  |
|  |  | ZMO1823 |  |
|  |  | ZMO1824 |  |
|  |  | ZMO1825 |  |
|  |  | ZMO1835 |  |
|  |  | ZMO1871 |  |
|  |  | ZMO1975 |  |
|  |  | ZMO1083 |  |
|  |  | ZMOp32x007 |  |
|  |  | ZMOp33x012 |  |
|  |  | ZMOp33x017 |  |
|  |  | ZMOp36x001 |  |

*Differences between ZmoMBEL601/*i*HN446 and *i*ZM363/*i*HN446 in genes.
