# Supplementary material for: A reconciliation of genome-scale metabolic network model of Zymomonas mobilis ZM4
Source: Sci Rep. 2020 May 8;10:7782. doi: 10.1038/s41598-020-64721-x (PMC7210963; doi:10.1038/s41598-020-64721-x)
Supplement: Supplementary file 4 — Supplementary Table S4. [file 41598_2020_64721_MOESM4_ESM.docx]

**A reconciliation of genome-scale metabolic network model of *Zymomonas mobilis* ZM4**

Hoda Nouri^1^, Hamideh Fouladiha^2^, Hamid Moghimi^*1^, Sayed-Amir Marashi^*2^

**S4 Table 2. Deleted/changed enzymes**

| **Deleted/changed enzymes** | | | |
| --- | --- | --- | --- |
| **ZmoMBEL601** | ***i*ZM363** | ***i*EM439** | ***i*HN446** |
| 1.3.3.1 | 1.3.3.1 |  | 1.3.98.1 |
| 4.2.1.52 | 4.2.1.52 |  | 4.3.3.7 |
| 5.4.2.1 | 5.4.2.1 | 5.4.2.1 | 5.4.2.11 |
| 1.1.1.158 | 1.1.1.158 |  | 1.3.1.98 |
| 2.3.1.41 |  |  | 2.3.1.38 |
| 2.5.1.- |  |  | 2.5.1.39 |
| 2.5.1.- |  |  | 2.5.1.90 |
| 1.3.99.1 | 1.3.99.1 | 1.3.99.1 | 1.3.5.1 |
| 1.3.1.26 | 1.3.1.26 |  | 1.17.1.8 |
| 2.7.8.- |  |  | 2.7.8.41 |
| 2.5.1.- |  |  | 2.5.1.78 |
| 4.1.1.- |  |  | 4.1.1.98 |
| 5.4.2.1 | 5.4.2.1 |  | 5.4.2.12 |
|  | 6.3.2.15 |  | 6.3.2.10 |
|  | 4.1.3.7 |  | 2.3.3.1 |

*changes in the biochemical data or genomic annotations have resulted in the changes in related enzymes.
